# Supplementary material for: Synthesis and Electrochemical Performance of Electrostatic Self-Assembled Nano-Silicon@N-Doped Reduced Graphene Oxide/Carbon Nanofibers Composite as Anode Material for Lithium-Ion Batteries
Source: Molecules. 2021 Aug 10;26(16):4831. doi: 10.3390/molecules26164831 (PMC8398711; doi:10.3390/molecules26164831)
Supplement: Supplementary file 1 [file molecules-26-04831-s001.zip › molecules-1278880-supplementary.pdf]

molecules

Supplementary Materials

Synthesis and Electrochemical Performance of Electrostatic Self-Assembled Nano-Silicon@N-Doped Reduced Graphene Oxide/Carbon Nanofibers Composite as Anode Material for Lithium-Ion Batteries

RuYe Cong [1], Hyun-Ho Park [1], Minsang Jo [2], Hochun Lee [2] and Chang-Seop Lee [1,*]

1 Department of Chemistry, Keimyung University, Daegu 42601, Korea; cry79838@naver.com (R.C.); rubchem@kmu.ac.kr (H.-H.P.)
2 Department of Energy Science and Engineering, DGIST, Daegu 42988, Korea; alstkdwh@dgist.ac.kr (M.J.); dukelee@dgist.ac.kr (H.L.)
* Correspondence: surfkm@kmu.ac.kr; Tel.: +82-53-580-5192

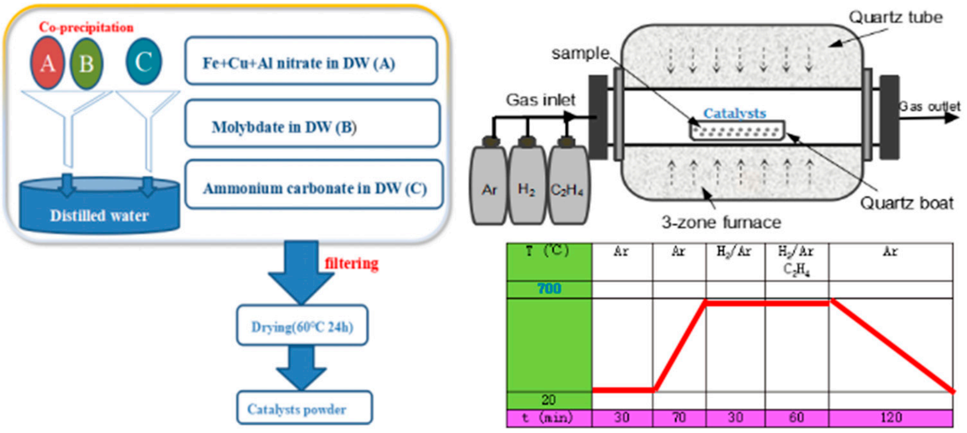

Figure S1. Process for preparing the catalysts and the synthesis of carbon nanofibers.

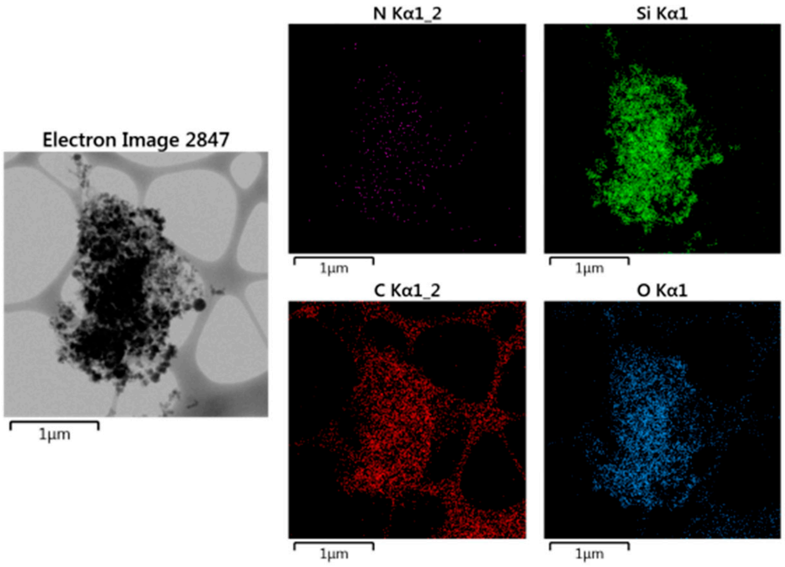

Figure S2. The EDS mapping of the Si, N, C, O elements on the surface of the Si@N-doped rGO/CNF composite.

Table S1. Raman spectroscopy results of the GO, rGO, CNFs, Si/rGO, and Si/CNF/rGO samples (see Figure 4(b)).

Sample	I_D/I_G
GO	1.05
rGO	0.96
CNF	0.99
Si@APTES/N-doped GO	1.03
Si/rGO	1.02
Si@N-doped rGO	1.00
Si@N-doped rGO/CNF	1.00

Table S2. Discharge capacity, coulomb efficiency, and capacity retention rate of the Si/CNF/rGO, Si@N-doped rGO and Si@N-doped rGO/CNF composite electrodes.

Samples	Max. Discharge Capacity (mAh/g)	Discharge Capacity (mAh/g)		Coulomb Efficiency (%)		Capacity Retention Rate (%)	
		After 37 Cycle	After 100 Cycle	First Cycle	100 Cycle	After 37 Cycle	After 100 Cycle
Si/CNF/rGO	3434.9	1054.2	964.7	51.7	99.9	30.7	26.5
Si@N-doped rGO	3138.8	1140.2	1091.8	66.1	99.5	36.3	34.8
Si@N-doped rGO/CNF	2192.3	1418.8	1276.8	71.5	99.9	64.7	58.2
